# Supplementary material for: Mutations in PCYT2 disrupt etherlipid biosynthesis and cause a complex hereditary spastic paraplegia
Source: Brain. 2019 Oct 22;142(11):3382–97. doi: 10.1093/brain/awz291 (PMC6821184; doi:10.1093/brain/awz291)
Supplement: awz291_Supplementary_Data [file awz291_supplementary_data.zip › awz291-Suppl_data/Supplementary_Data4.zip!/Supplementary information 4 - plasma lipidomics.zip!/Plasma lipidomics/Control vs Patient1/Statistical plots and data/DG species/Volcano plot_alternative.pdf]

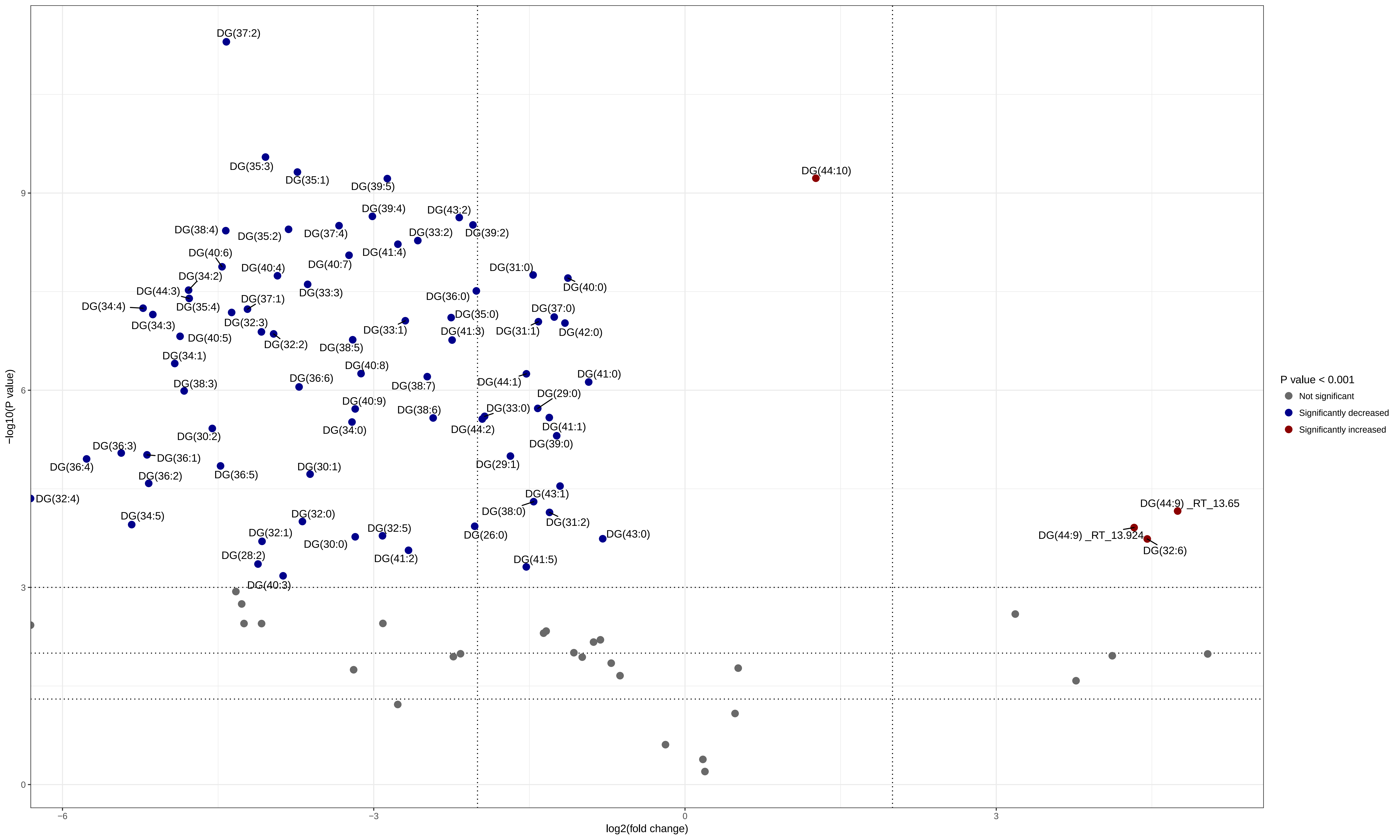
DG(37:2)
DG(35:3)
DG(35:1)
DG(39:5)
DG(44:10)
DG(39:4)
DG(43:2)
DG(38:4)
DG(35:2)
DG(37:4)
DG(33:2)
DG(39:2)
DG(40:6)
DG(41:4)
DG(40:4)
DG(40:7)
DG(31:0)
DG(34:2)
DG(40:0)
DG(44:3)
DG(33:3)
DG(36:0)
DG(34:4)
DG(35:4)
DG(37:1)
DG(35:0)
DG(37:0)
DG(34:3)
DG(32:3)
DG(33:1)
DG(41:3)
DG(31:1)
DG(42:0)
DG(40:5)
DG(32:2)
DG(38:5)
DG(34:1)
DG(40:8)
DG(36:6)
DG(41:0)
DG(38:3)
DG(38:7)
DG(44:1)
DG(29:0)
DG(40:9)
DG(38:6)
DG(33:0)
DG(41:1)
DG(34:0)
DG(44:2)
DG(30:2)
DG(39:0)
DG(36:3)
DG(36:1)
DG(29:1)
DG(36:4)
DG(30:1)
DG(36:2)
DG(36:5)
DG(43:1)
DG(32:4)
DG(44:9) _RT_13.65
DG(38:0)
DG(34:5)
DG(32:0)
DG(31:2)
DG(32:1)
DG(32:5)
DG(26:0)
DG(43:0)
DG(44:9) _RT_13.924
DG(30:0)
DG(32:6)
DG(28:2)
DG(41:2)
DG(41:5)
DG(40:3)
−log10(P value)
9
6
3
0
−6
−3
0
3
log2(fold change)
P value < 0.001
Not significant
Significantly decreased
Significantly increased
